# Cyclin-dependent kinase 1-mediated phosphorylation of SET at serine 7 is essential for its oncogenic activity

**DOI:** 10.1038/s41419-019-1621-2

**Published:** 2019-05-16

**Authors:** Ling Yin, Yongji Zeng, Yi Xiao, Yuanhong Chen, Hong Shen, Jixin Dong

**Affiliations:** 1 Department of Oncology, Xiangya Hospital, Central South University, 410008 Changsha, China; 2 Eppley Institute for Research in Cancer and Allied Diseases, Fred & Pamela Buffett Cancer Center, University of Nebraska Medical Center, Omaha, NE 68198 USA; 3 Department of Biochemistry and Molecular Biology, Shandong University School of Basic Medical Science, 250012 Jinan, China

**Keywords:** Cell growth, Cell signalling

## Abstract

SE translocation (SET), an inhibitor of protein phosphatase 2A (PP2A), plays important roles in mitosis and possesses oncogenic activity in several types of cancer. However, little is known regarding its regulation. Here we reveal a novel phosphorylation site of SET isoform 1, and we have determined its biological significance in tumorigenesis. We found that the mitotic kinase cyclin-dependent kinase 1 (CDK1) phosphorylates SET isoform 1 in vitro and in vivo at serine 7 during antitubulin drug-induced mitotic arrest and normal mitosis. SET deletion resulted in massive multipolar spindles, chromosome misalignment and missegregation, and centrosome amplification during mitosis. Moreover, mitotic phosphorylation of SET isoform 1 is required for cell migration, invasion, and anchorage-independent growth in vitro and tumorigenesis in xenograft animal models. We further documented that SET phosphorylation affects Akt activity. Collectively, our findings suggest that SET isoform 1 promotes oncogenesis in a mitotic phosphorylation-dependent manner.

## Introduction

SET, also known as inhibitor 2 of protein phosphatase 2A (I2PP2A), directly binds to PP2A and inhibits its phosphatase activity^1–3^. PP2A plays a critical role in neoplastic transformation by negatively regulating many oncogenic signaling pathways, and it serves as a therapeutic target^4,5^. Accumulated evidence demonstrated that SET functions as an oncogene in several neoplasms and promotes tumorigenesis^6,7^. Overexpression of SET has been shown in breast cancer^8^, ovarian cancer^9^, leukemia^10,11^, lymphoma^12^, hepatocellular carcinoma^13^, prostate cancer^14^, colon cancer^15^, non-small-cell lung carcinoma^16^, and pancreatic cancer^17^. Furthermore, upregulation of SET correlates with poor clinical outcomes in hepatocellular carcinoma, ovarian cancer, and colorectal cancer^13,15,18^. In line with these clinical observations, SET silencing or pharmacological inhibition of SET significantly impaired tumor growth in various human malignancies^6^. Interestingly, ectopic expression of SET also promoted resistance to chemotherapeutics in colon cancer, nonsmall cell lung carcinoma, and hematologic malignancies^10,11,13,15,19^. Promisingly, several antagonists (e.g., FTY720, OP449, and EMQA) that target the SET–PP2A interface have been shown to inhibit tumor growth and overcome therapeutic resistance in various preclinical models^10,11,20^. In addition to its oncogenic roles, SET is also involved in various other molecular processes, including histone modification, chromatin remodeling, DNA repair, gene transcription, and androgen synthesis^6,7,9^. Mechanistically, SET has been shown to interact with several PP2A-regulated oncogenic pathways, including the Akt, mitogen-activated protein kinase, and BCR-ABL pathways^6,10^. However, SET may regulate cellular physiology through PP2A-independent pathways.

Although extensive studies have demonstrated the important roles for SET-PP2A signaling in tumorigenesis, the underlying mechanisms are less clear. Mitotic aberrations cause aneuploidy or genomic instability, which is a hallmark of human malignancy^21,22^. Therefore, mitosis has been a long-standing anticancer drug target^23–26^. Interestingly, SET has been linked to the mitotic machinery. For example, SET associates with cyclin B and inhibits cyclin B-CDK1 activity^27,28^. Knockdown (KD) of SET delayed mitotic progression and inhibited G2/M transition^29^. Moreover, primarily located in the nucleus, SET has been demonstrated to protect histones from acetylation, modulation of chromosome condensation, and cohesion^30^. Overexpression of SET resulted in precocious separation of chromatids in mouse oocytes^31^. SET is centromere localized^29,31^ and forms a complex with linker histones and shugoshins during mitosis^29^. Several studies demonstrated that SET mediates timely resolution of sister chromatids during mitosis and functions as a mitotic chaperone^29,32^. These studies suggest that SET might exert its oncogenic function through dysregulation of mitosis.

The human SET gene is located on chromosome 9q34 and contains 11 exons and 10 introns. It was originally identified as part of a fusion gene with nucleoporin Nup214 (CAN) in a patient with acute undifferentiated leukemia in 1992^33,34^. There are four protein isoforms of SET and the only difference is in the first exon among them^17,35^. Isoforms 1 and 2 are the most thoroughly characterized ones^17^. SET is a phosphoprotein, and two sites (S9 and S24 in isoform 2) have been identified as phosphorylated by protein kinase C in vivo^33^. Furthermore, casein kinase 2-mediated phosphorylation at S9 causes cytoplasmic retention of SET and induces hyperphosphorylation of tau in Alzheimer disease^36,37^. However, the regulation of SET in mitosis and its possible role in cancer have remained unknown. Given the critical roles of SET in mitosis and oncogenesis, we sought to elucidate the regulatory mechanisms of SET in mitosis. We found that the mitotic kinase cyclin-dependent kinase 1 (CDK1) phosphorylates SET at S7 (isoform 1) during mitosis. Moreover, we showed that mitotic phosphorylation of SET is required for precise mitosis and oncogenic activity.

## Materials and methods

### Expression constructs

The full-length human SET cDNA clone (isoform 1, NM_001122821.1) was purchased from GeneCopoeia (EX-Z5816-M02-B, Rockville, MD, USA). A point mutation (Serine 7 to Alanine) was generated by the QuikChange Site-Directed PCR Mutagenesis Kit (Stratagene, La Jolla, CA, USA) and verified by sequencing. To make the lentivirus-mediated SET expression construct, the above cDNA was cloned into the pSIN4-Flag-IRES-neo vector. The pSIN4-Flag-IRES-neo vector was made by replacing the puromycin-coding sequence of the pSIN4-Flag-IRES-puro vector^38^ with a neomycin-coding sequence.

### Cell culture

HEK293T, HeLa, and RKO cell lines were purchased from American Type Culture Collection (ATCC, Manassas, VA, USA). The cell lines were authenticated at ATCC and were used at low (<20) passages. All other cell lines were maintained in Dulbecco’s modified Eagle’s medium supplemented with 10% FBS. HPNE (an immortalized human pancreatic nestin-expressing cell line) was kindly provided by Dr Michel Ouellette (University of Nebraska Medical Center), who originally established the cell line and deposited it at ATCC^39^. Attractene (Qiagen, Germantown, MD, USA) was used for transient overexpression transfections following the manufacturer’s instructions. Nocodazole (100 ng/ml for 16–20 h) and Taxol (100 nM for 16–20 h) were used to arrest cells at late G2 and prometaphase (G2/M), unless otherwise indicated. VX680 (Aurora-A, -B, -C inhibitor), BI2536 (Plk1 inhibitor), and MK2206 (Akt inhibitor) were purchased from Selleck Chemicals (Houston, TX, USA). U0126 (MEK1/2 inhibitor), SB203580 (p38 inhibitor), LY294002 (PI-3K inhibitor), rapamycin (mTOR inhibitor), and SP600125 (JNK1/2 inhibitor) were from LC Laboratories (Woburn, MA, USA). RO3306 (CDK1 inhibitor) and Purvalanol A (CDK1/2/5 inhibitor) were from ENZO Life Sciences (Farmingdale, NY, USA). MK5108 (Aurora-A inhibitor) and SB216763 (GSK3β inhibitor) were from Merck (Kenilworth, NJ, USA) and Sigma-Aldrich (Burlington, MA, USA), respectively.

### Cell line establishment

Stable overexpression and re-expression of SET (wild-type and S7A mutant) in SET-KD cells were achieved by lentivirus-mediated infection and selection^40^. Gene KD was achieved by an shRNA-mediated method. The MISSION shRNA plasmids targeting human SET were purchased from Sigma-Aldrich (TRCN0000063717). To make the shRNA-resistant (Res) SET cDNA, the target sequence (5′-CCACCGAAATCAAATGGAAATCT-3′) was changed into 5′-CaACtGAgATCAAATGGAAATCT-3′ by PCR mutagenesis. The mutated SET cDNA was then cloned into the pSIN4-Flag-IRES-Neo vector to generate a Flag-tagged shRNA-resistant SET construct.

### Recombinant protein purification and in vitro kinase assay

The glutathione S-transferase (GST)-tagged proteins were bacterially expressed and purified on GSTrap FF affinity columns (GE Healthcare, Chicago, IL, USA) following the manufacturer’s instructions. About 0.5 µg of GST-SET proteins were incubated with 10 U recombinant CDK1/cyclin B1 complex (New England Biolabs, Ipswich, MA, USA) in the presence of 5 µCi γ-^32^P-ATP (3000 Ci/mmol, PerkinElmer, Waltham, MA, USA). Purified CDK1/cyclin B1 complex from SignalChem (Richmond, BC, Canada) was also used for in vitro kinase assays using phospho-specific antibodies. The samples were resolved by SDS-PAGE, transferred onto PVDF (Millipore, Burlington, MA, USA), and visualized by autoradiography or detected by phospho-specific antibodies.

### Antibodies

The anti-SET monoclonal antibody from Santa Cruz Biotechnology (SC133138, Dallas, TX, USA) was used throughout the study. Rabbit polyclonal phospho-specific antibody against human SET S7 (isoform 1) was generated and purified by AbMart (Shanghai, China). The peptide for generating the phospho antibody is: APKRQ-pS-PLPPQ. The corresponding non-phospho-peptide was also synthesized for antibody purification. Anti-Flag antibodies were from Sigma-Aldrich. Anti-β-actin, anti-ERK1/2, anti-phospho-S380 RSK, anti-MAD1, anti-MAD2, anti-TTK, anti-CDC27, anti-CDC20, and anti-cyclin B1 antibodies were from Santa Cruz Biotechnology. Anti-Aurora-A, anti-Bub1, anti-BubR1, anti-Cyclin C, and anti-GST antibodies were from Bethyl Laboratories (Montgomery, TX, USA). The following antibodies were obtained from Cell Signaling Technology (Danvers, MA, USA): Phospho-T288/T232/T198 Aurora-A/B/C, phospho-S10 H3, phospho-T202/Y204 ERK1/2, phospho-S127 YAP, phospho-S397 YAP, Yes-associated protein (YAP), ribosomal S6 kinase 1 (RSK1), phospho-S807/S811 Rb, Rb, phospho-S536 NF-kB p65, NF-kB p65, phospho-S176/S180 IKKα/β, IKKα/β, phospho-T308 Akt, phospho-S473 Akt, Akt, phospho-Y705 STAT3, signal transducers and activators of transcription 3 (STAT3), phospho-S727 STAT1, phospho-Y1034/Y1035 JAK1, Janus kinase 1 (JAK1), phospho-S33/S37/T41 β-catenin, β-catenin, CDK1, CDK3, CDK4, CDK5, Cyclin A2, Cyclin D1, Cyclin E1, p21, and CDC25C. Anti-β-tubulin (Sigma-Aldrich) and anti-γ-tubulin (Biolegend, San Diego, CA, USA) antibodies were used for immunofluorescence staining.

### Phos-tag and western blot analysis

Phos-tag^TM^ SDS-acrylamide gels were used as we described previously^41^. Western blotting, immunoprecipitation, and lambda phosphatase treatment assays were done as described^40^.

### Immunohistochemistry (IHC) staining, immunofluorescence staining, and confocal microscopy

Fluorescence staining and confocal microscopy were done, as previously described^42^. IHC staining (of Ki-67, cleaved caspase 3, and p-T308 Akt) in tumor tissues was performed according to protocols described^43,44^. Anticleaved caspase 3 (1:100) and p-T308 Akt (1:100) antibodies were from Cell Signaling Technology. Anti-Ki-67 antibody was from Thermo Scientific (Waltham, MA, USA) and was used at 1:100 dilutions.

### Cell proliferation, colony formation, migration, and invasion assays

Cell proliferation and colony formation assays in soft agar (anchorage-independent growth) were performed as described^44,45^. In vitro analysis of invasion and migration (1.0 × 10^5^ cells) was assessed using the BioCoat invasion system (BD Biosciences, Franklin Lakes, NJ, USA) and Transwell system (Corning, Corning, NY, USA), respectively, according to the manufacturer’s instructions. The invasive and migratory cells were stained with 0.1% crystal violet and counted manually.

### Animal studies

For in vivo xenograft studies, RKO cells (1.0 × 10^6^ cells each line) were subcutaneously injected into both flanks of 6-week-old male athymic nude mice (Ncr-nu/nu, Harlan, Indianapolis, IN, USA). Cells were suspended in phosphate-buffered saline (PBS) and mixed with Matrigel in 1:1 ratio (volume). Five animals were used per group. Tumor sizes were measured twice a week using an electronic caliper 10 days post injection. Tumor volume (*V*) was calculated by the formula: *V* = 0.5 × length × width^2,^^45^. Mice were euthanized by CO_2_ inhalation at the end of the experiment and the tumors were excised for subsequent analysis. The animals were housed in pathogen-free facilities. All animal experiments were approved by the University of Nebraska Medical Center Institutional Animal Care and Use Committee.

### Statistical analysis

Statistical significance was performed using a two-tailed, unpaired Student’s *t* test.

## Results

### SET is phosphorylated during antitubulin drug-induced mitotic arrest

**Fig. 1:**
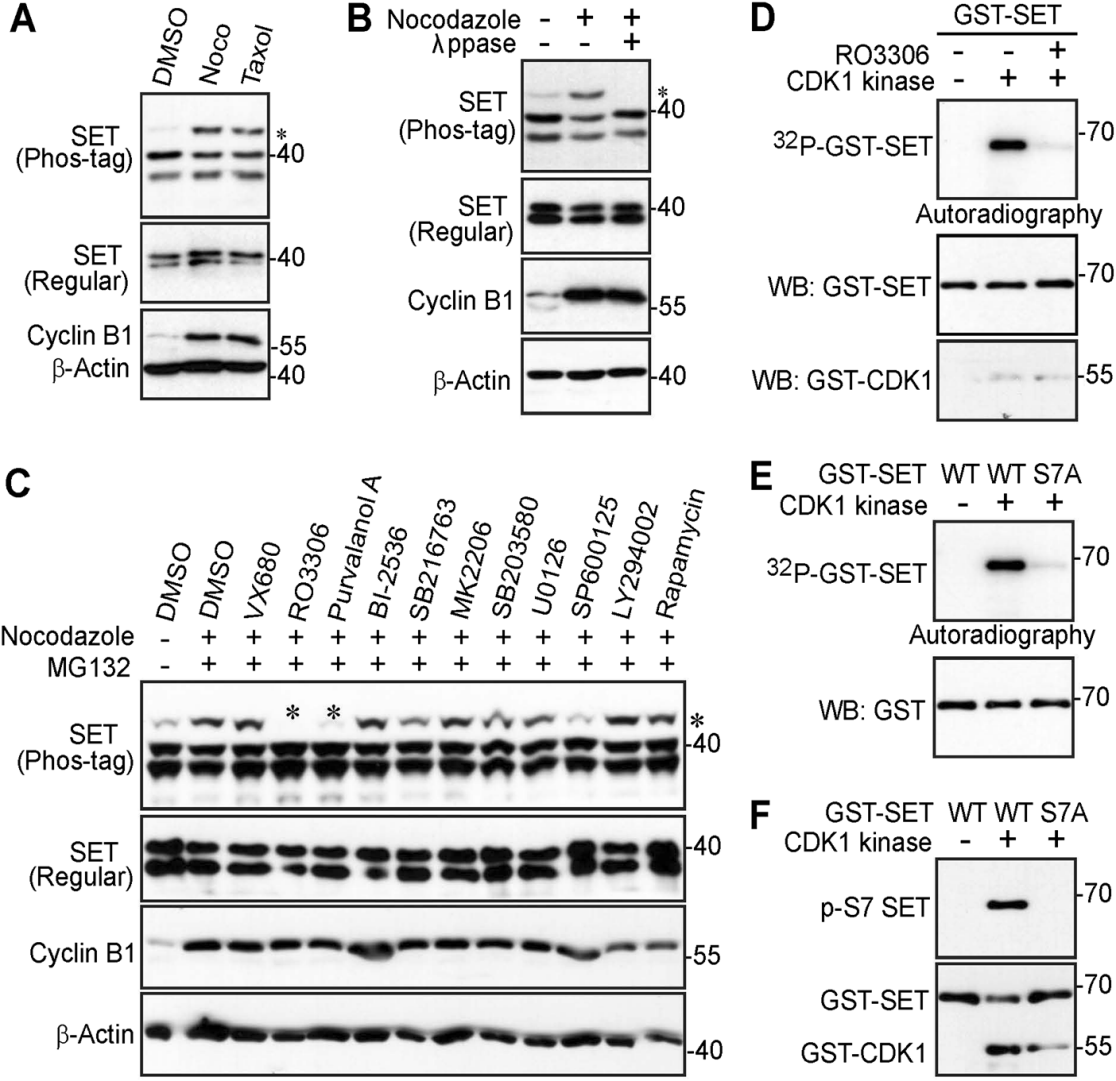
CDK1/cyclin B1 kinase complex phosphorylates SET isoform 1 in vitro. **a** HeLa cells were treated with DMSO (control), taxol (100 nM for 16 h), or nocodazole (Noco, 100 ng/ml for 16 h). Total cell lysates were electrophoresed on regular and Phos-tag SDS polyacrylamide gels and probed with the indicated antibodies. Increased cyclin B1 levels marks cells in mitosis. An asterisk (*) marks the phosphorylated/shifted band. **b** HeLa cells were treated with nocodazole as indicated and cell lysates were further treated with (+) or without (−) λ phosphatase (ppase). Total cell lysates were probed with the indicated antibodies. Increased cyclin B1 levels marks cells in mitosis. An asterisk marks the phosphorylated/shifted band. **c** HeLa cells were treated with nocodazole, with or without various kinase inhibitors as indicated. Inhibitors were added 1.5 h before harvesting the cells (with MG132 to prevent cyclin B degradation and subsequent mitotic exit). The concentrations used for each inhibitor were as follows: VX680 2 μM, RO3306 5 μM, Purvalanol A 10 μM, BI-2536 100 nM, SB216763 10 μM, MK-2206 10 μM, SB203580 10 μM, U0126 20 μM, SP600125 20 μM, LY294002 30 μM, and rapamycin 100 nM. Total cell lysates were electrophoresed on regular and Phos-tag SDS polyacrylamide gels and probed with the indicated antibodies. Increased cyclin B1 levels mark cells in mitosis. An asterisk marks the phosphorylated/shifted band. **d** In vitro kinase assays with purified CDK1/cyclin B1 complex using GST-tagged SET isoform 1 proteins as substrates. RO3306 (5 µM) was used to inhibit CDK1/cyclin B1 kinase activity. **e** GST-SET and GST-SET-S7A proteins were used for in vitro kinase assays with purified CDK1/cyclin B1 complex. **f** In vitro kinase assays were done as in **e** except anti-phospho-SET S7 antibody was used

To explore the phospho status of SET during mitosis, we treated HeLa cells with taxol or nocodazole (both agents arrest cells in prometaphase after an overnight treatment) and examined the response of SET on a Phos-tag gel. SET proteins were shown as a doublet (isoform 1 and isoform 2) on an SDS-PAGE gel (Fig. 1a). Interestingly, a significant portion of SET protein was upshifted/retarded on a Phos-tag gel during mitotic arrest, suggesting that SET is phosphorylated under these conditions. Lambda phosphatase treatment eliminated the slow-migrating band (the top band on the gel), indicating that the mobility shift of SET during mitotic arrest is caused by phosphorylation (Fig. 1b). The middle and bottom bands remained unchanged during phosphatase treatment (Fig. 1b).

### Identification of the corresponding kinase for SET isoform 1 phosphorylation

In order to determine which upstream kinase(s) could be responsible for SET phosphorylation, we treated cells with various kinase inhibitors together with MG132 (stabilizes cyclin B1 and prevent cells from exiting mitosis). Interestingly, the most significant inhibition of phosphorylation of SET was observed in cells treated with RO3306 (a CDK1 inhibitor) and Purvalanol A (inhibits CDK1/2/5) (Fig. 1c), suggesting that CDK1, a well-known mitotic kinase, is the candidate kinase for SET phosphorylation. Taken together, these data suggest that mitotic arrest-induced SET phosphorylation is CDK1 dependent.

### CDK1 phosphorylates SET isoform 1 in vitro

Next, we performed in vitro kinase assays with GST-tagged SET proteins as substrates to determine whether CDK1 kinase can directly phosphorylate SET. Figure 1d shows that purified CDK1/cyclin B1 complex phosphorylated GST-SET in vitro (Fig. 1d). As expected, addition of RO3306 abolished the ^32^P incorporation into SET (Fig. 1d).

CDK1 phosphorylates an S/TP consensus sequence^46^. Database analysis (www.phosphosite.org) identified serine 7 (followed by a proline) as a possible phosphorylation site in SET during mitosis^47^. Of interest, mutating S7 to alanine largely eliminated the phosphorylation (^32^P incorporation) of SET (Fig. 1e), suggesting that S7 is the main phosphorylation site of SET in vitro. Next, we generated a phospho-specific antibody against SET S7. Using this antibody, we confirmed that GST-SET proteins were robustly phosphorylated at S7 by CDK1/cyclin B1 kinase complex in vitro (Fig. 1f).

### SET isoform 1 is phosphorylated at S7 in cells in a CDK1-dependent manner

**Fig. 2:**
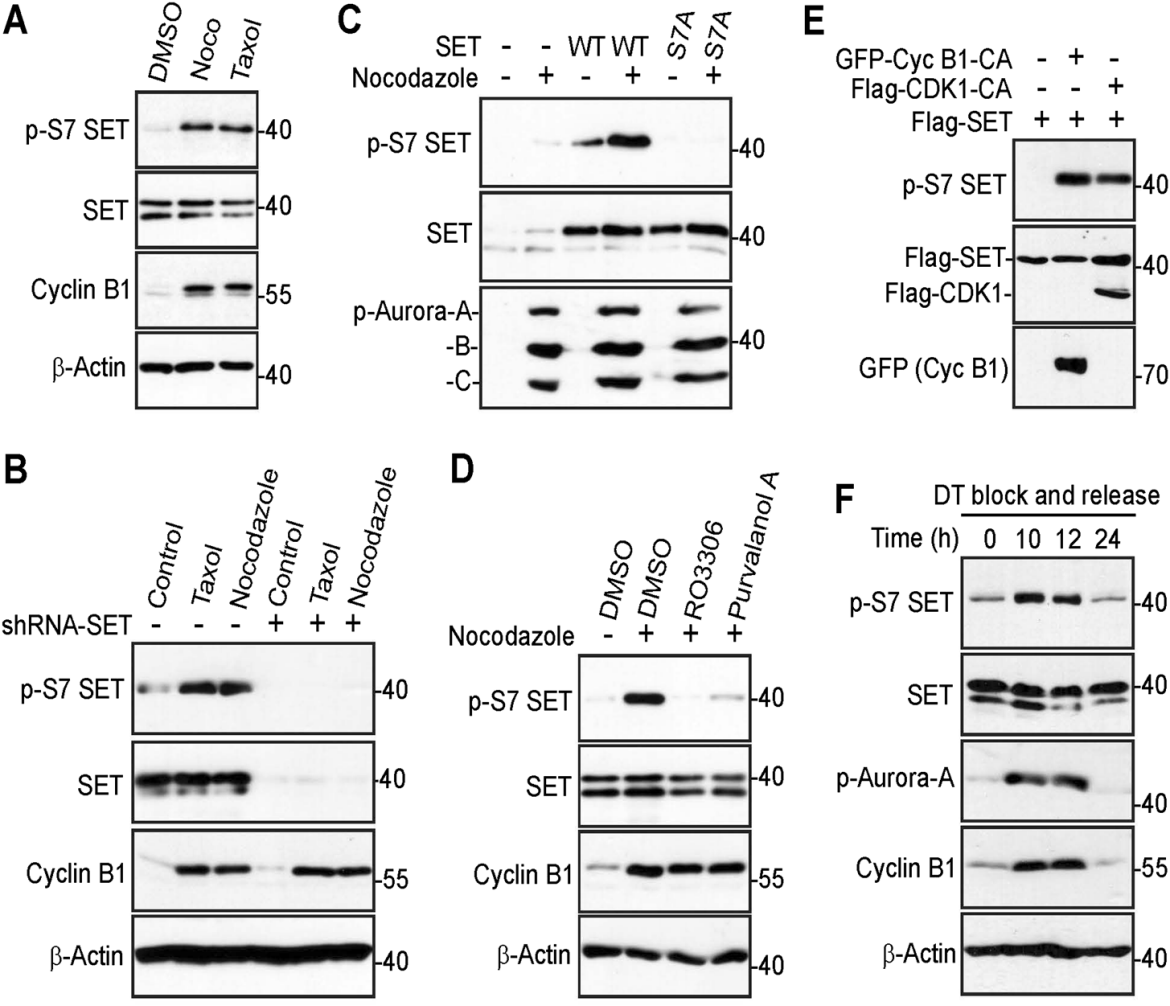
CDK1 phosphorylates SET isoform 1 at S7 in cells. **a** HeLa cells were treated with DMSO, nocodazole (Noco), or taxol and total cell lysates were probed with the indicated antibodies. Increased cyclin B1 levels mark cells in mitosis. **b** Control and SET-knockdown (both isoforms 1 and 2) HeLa cells were subjected to the indicated treatment. Total cell lysates were analyzed by western blotting with the indicated antibodies. Increased cyclin B1 levels mark cells in mitosis. **c** HEK293T cells were transfected with isoform 1 SET or SET-S7A plasmid. At 30 h posttransfection, the cells were treated with nocodazole for 16 h. Total cell lysates were probed with the indicated antibodies. Increased p-Aurora A/B/C levels mark cells in mitosis. **d** HeLa cells were treated with nocodazole for 16 h and RO3306 (5 µM) or Purvalanol A (10 µM) was added to cells 1.5 h before harvesting as indicated. Proteasome inhibitor MG132 was also added (together with inhibitors) to prevent cyclin B from degradation and cells from exiting from mitosis. Increased cyclin B1 levels mark cells in mitosis. **e** HEK293T cells were transfected with expression constructs as indicated and total cell lysates were analyzed by western blotting. GFP-Cyc B1-CA: GFP-Cyclin B1-R42A (a nondegradable/constitutive active mutant). Flag-CDK1-CA: Flag-CDK1-T14A/Y15A (nonphosphorylatable/constitutive active CDK1). **f** HeLa cells were synchronized by a double thymidine (DT) block and release method. Total cell lysates were harvested at the indicated time points and subjected to western blotting analysis. Increased p-Aurora-A and cyclin B1 levels mark cells in mitosis

After confirming SET phosphorylation at S7 by CDK1 in vitro, we next examined this phosphorylation in cells. Nocodazole or taxol treatment significantly increased phosphorylation of S7 of endogenous SET (Fig. 2a). The shRNA-mediated depletion of SET (both isoform 1 and isoform 2) largely blocked the phospho signal, confirming the specificity of the phospho antibody (Fig. 2b). Furthermore, nocodazole treatment significantly increased the phosphorylation of S7 of transfected SET isoform 1, and the signal was abolished by mutating S7 to alanine (Fig. 2c). Treatment with kinase inhibitors RO3306 or Purvalanol A greatly decreased phosphorylation of S7 induced by nocodazole, suggesting that phosphorylation of SET S7 is CDK1 kinase dependent (Fig. 2d). Consistent with these observations, an enhanced expression of constitutive active CDK1 or cyclin B1 was sufficient to stimulate SET phosphorylation at S7 (Fig. 2e). Taken together, these results indicate that SET isoform 1 is phosphorylated at S7 in cells during mitotic arrest in a CDK1-dependent manner.

To determine whether mitotic phosphorylation at SET isoform 1 S7 occurs during normal mitosis, we determined the phospho status of cells collected from a double thymidine block and release^42^. After being released from double thymidine block, cells enter into mitosis at 10–12 h, as revealed by increased p-S10 H3 levels^41,48^. We found that the p-SET S7 signal was coincidently increased in these cells (Fig. 2f). These observations suggest that SET isoform 1 is phosphorylated at S7 during mitosis.

### Mitotic phosphorylation of SET isoform 1 is required for precise mitosis

**Fig. 3:**
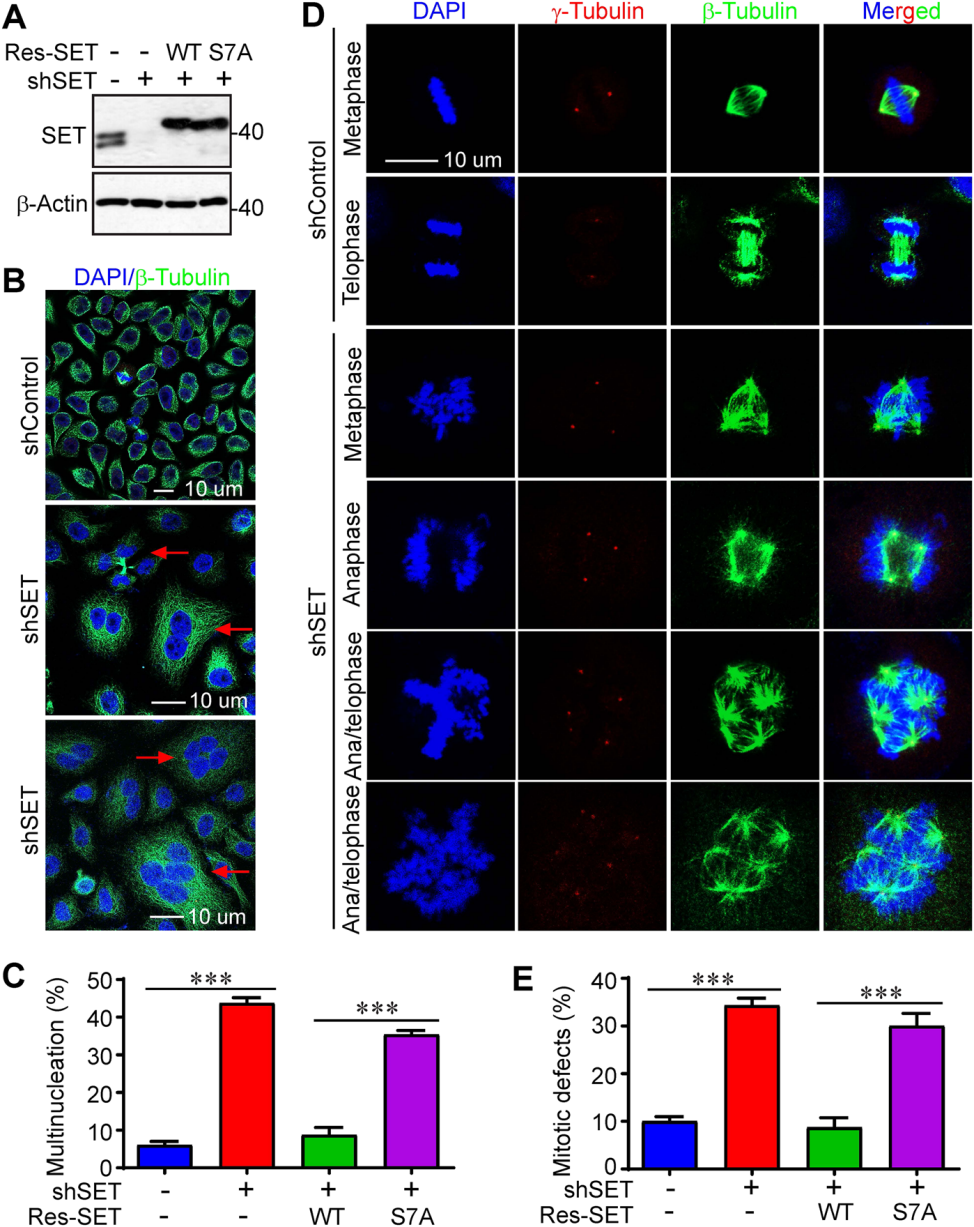
Phosphorylation of SET isoform 1 at S7 is required for proper mitosis in HeLa cells. **a** Western blotting analysis of HeLa SET-knockdown cells stably expressing Flag-tagged shRNA-resistant (Res) isoform 1 SET-WT or SET-S7A. **b**, **c** Knockdown of SET resulted in multinucleated cells. Representative photos of control (shControl) and SET-knockdown (shSET) cells were shown in **b**. Quantification of multinucleated cells (**c**) in cell lines established in **a**. Arrows in **b** indicate some of the multinucleated cells. Data in **c** were expressed as the mean ± SD of three independent experiments. ***p < 0.001 (unpaired Student’s *t* test). A total of 250 cells in each group were counted. **d**, **e** Knockdown of SET resulted in massive mitotic defects. Representative photos of normal mitosis (shControl) and mitotic abnormalities (shSET) in HeLa cells were shown. Cells were stained with β-tubulin, γ-tubulin antibodies, and DAPI to visualize microtubules (green), centrosomes (red), and chromosomes (blue), respectively. Data in **e** were collected from 160 mitotic cells for each group (mean ± SD of three independent experiments). ***p < 0.001 (unpaired Student’s *t* test)

SET has been shown to be involved in mitotic progression and its alteration resulted in mitotic defects^27,29^. To determine the functional significance of S7 phosphorylation, we established cell lines expressing shRNA-resistant SET or SET-S7A in SET-KD cells (Fig. 3a). Consistent with previous studies, we found that KD of SET (both isoform 1 and isoform 2) in HeLa cells resulted in a significant higher percentage of cells with multiple nuclei, suggesting a role of SET in cytokinesis (Fig. 3b, c). This phenotype was largely rescued by re-expressing wild-type SET in SET-KD cells, and the phospho-deficient mutant (SET-S7A) failed to restore this defect, suggesting that mitotic phosphorylation at S7 is essential for mitotic cell division (Fig. 3c).

We observed largely normal chromosomal alignment/segregation (DAPI staining), microtubule/spindle formation (immunofluorescence staining with β-tubulin), and centrosome number (γ-tubulin staining) during mitosis in control cells (Fig. 3d). In contrast, massive mitotic defects (e.g., multipolar spindles, supernumerary centrosomes, and chromosome misalignment/missegregation) were detected in SET-KD cells (Fig. 3d, e). Again, re-expression of wild-type SET, but not the SET-S7A mutant, completely rescued the mitotic defects in SET-KD cells (Fig. 3e). These data suggest that mitotic phosphorylation of SET isoform 1 is required for precise mitosis in HeLa cells.

### Mitotic phosphorylation of SET isoform 1 is required for cell migration and invasion

**Fig. 4:**
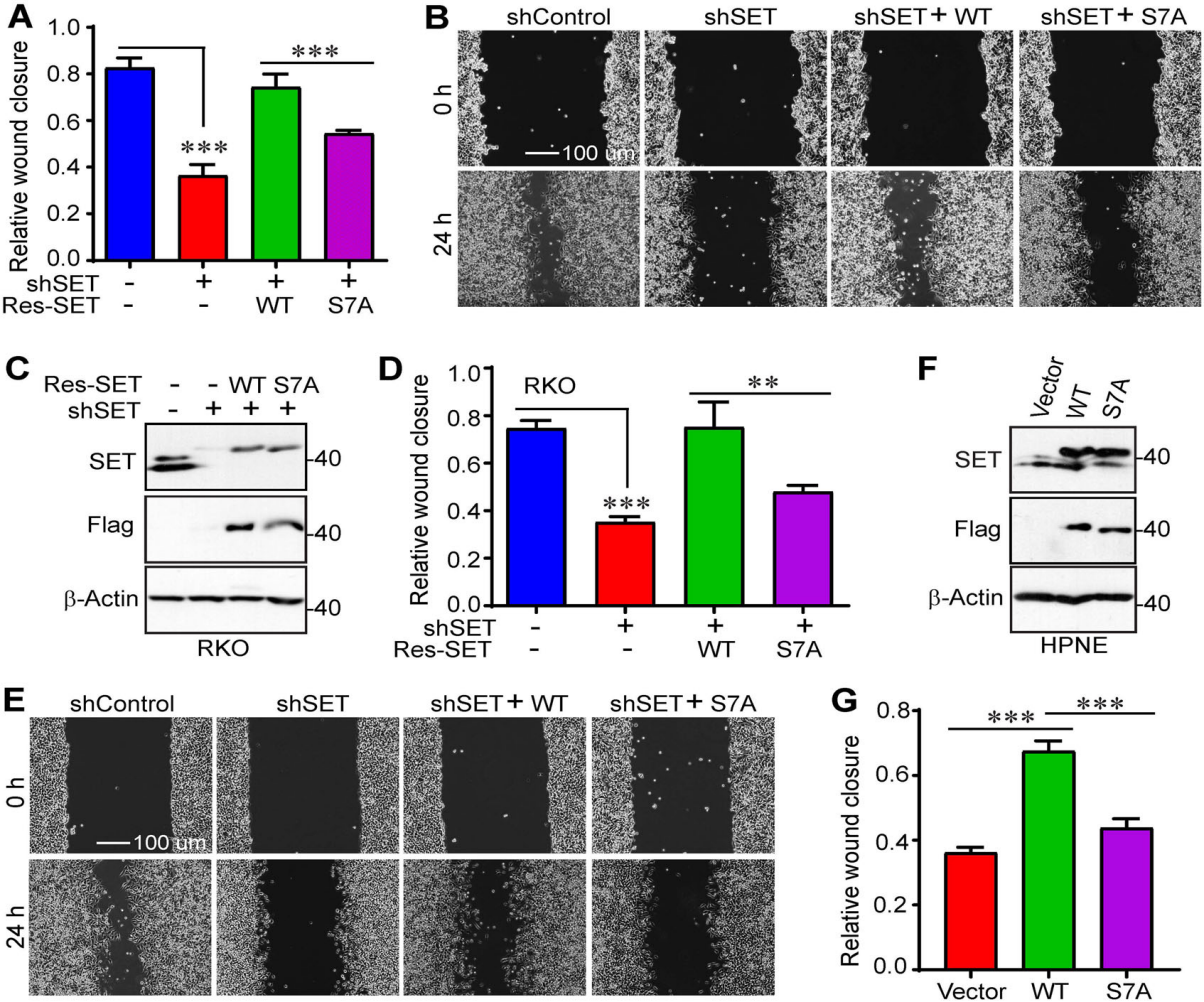
Mitotic phosphorylation of SET isoform 1 promotes cell migration. **a**, **b** Cell migration (wound healing) assays with cell lines established in Fig. 3a. Data were expressed as the mean ± SD of three independent experiments. ***p < 0.001 (unpaired Student’s *t* test). **c**–**e** Cell migration (wound healing) assays in RKO cells. Data were expressed as the mean ± SD of three independent experiments. ***p < 0.001; **p < 0.01 (unpaired Student’s *t* test). **f**, **g** Cell migration (wound healing) assays in immortalized human pancreatic nestin-expressing (HPNE) cells. Data were expressed as the mean ± SD of four independent experiments. ***p < 0.001 (unpaired Student’s *t* test)

Next, we further explored the biological significance of mitotic phosphorylation of SET in cancer cell growth. First, wound scratch assays were performed in control, SET-KD, and SET-KD cells expressing SET or SET-S7A. As expected, KD of SET (both isoforms 1 and 2) significantly decreased the rate of wound closure for HeLa cells (Fig. 4a, b). Similar results were obtained with colon cancer RKO cells (relatively high expression of SET) (Fig. 4c–e). Exogenous expression of wild-type SET, but not SET-S7A, restored migration of both HeLa and RKO cells (Fig. 4a–e). In line with loss-of-function phenotypes, ectopic expression of SET isoform 1 substantially increased the rate of wound closure in immortalized human pancreatic cells (HPNE, express relatively low SET) (Fig. 4f, g). The ability to increase wound closure for the isoform 1 SET-S7A mutant was greatly reduced when compared with wild-type SET isoform 1 (Fig. 4g).

**Fig. 5:**
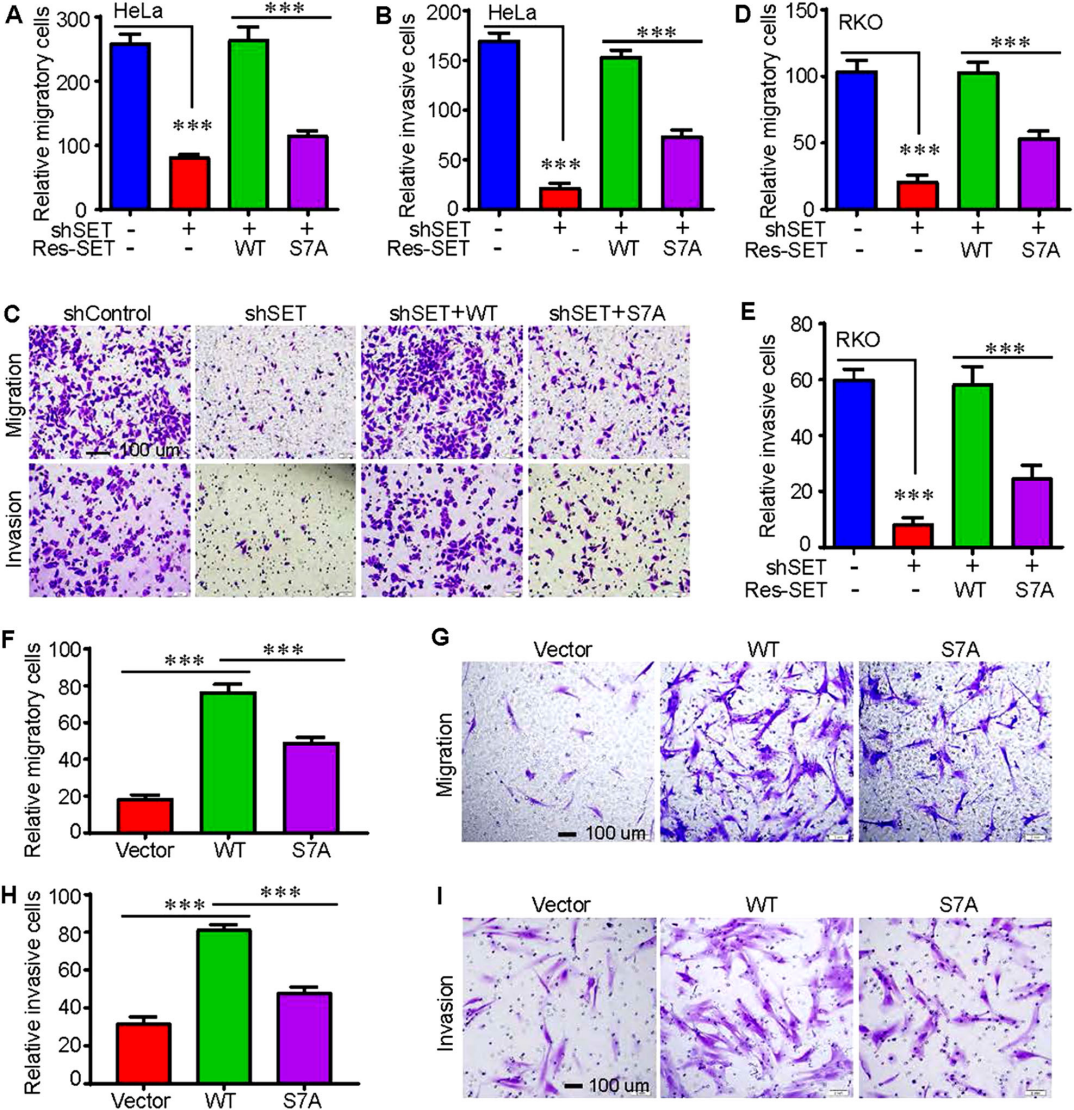
Mitotic phosphorylation of SET isoform 1 promotes cell migration and invasion. **a**–**c** Cell migration (Transwell) and invasion (Matrigel) assays with cell lines established in Fig. 3a (HeLa). Data were expressed as the mean ± SD of three independent experiments. Migratory and invasive cells were stained with crystal violet and representative fields were shown in **c**. ***p < 0.001 (unpaired Student’s *t* test). **d**, **e** Cell migration (Transwell) and invasion (Matrigel) assays in RKO cells established in Fig. 4c. Data were expressed as the mean ± SD of three independent experiments. ***p < 0.001 (unpaired Student’s *t* test). **f**–**i** Cell migration (Transwell) and invasion (Matrigel) assays in HPNE cells established in Fig. 4f. Data were expressed as the mean ± SD of four independent experiments. ***p < 0.001 (unpaired Student’s *t* test)

Second, we used Transwell and Matrigel systems to examine the migratory and invasive properties of these cells. Consistent with the data from Fig. 4, re-expression of wild-type SET isoform 1 completely rescued migration and invasion in HeLa and RKO cells (Fig. 5a–e). However, expression of the nonphosphorylatable mutant SET-S7A only modestly restored these characteristics (Fig. 5a–e). Enhanced expression of wild-type SET, and SET-S7A to a significantly lesser extent, promoted migratory and invasive abilities of HPNE cells (Fig. 5f–i). These observations suggest that mitotic phosphorylation of SET isoform 1 at S7 is essential for cell motility.

### Mitotic phosphorylation of SET isoform 1 is required for cell proliferation and anchorage-independent growth

**Fig. 6:**
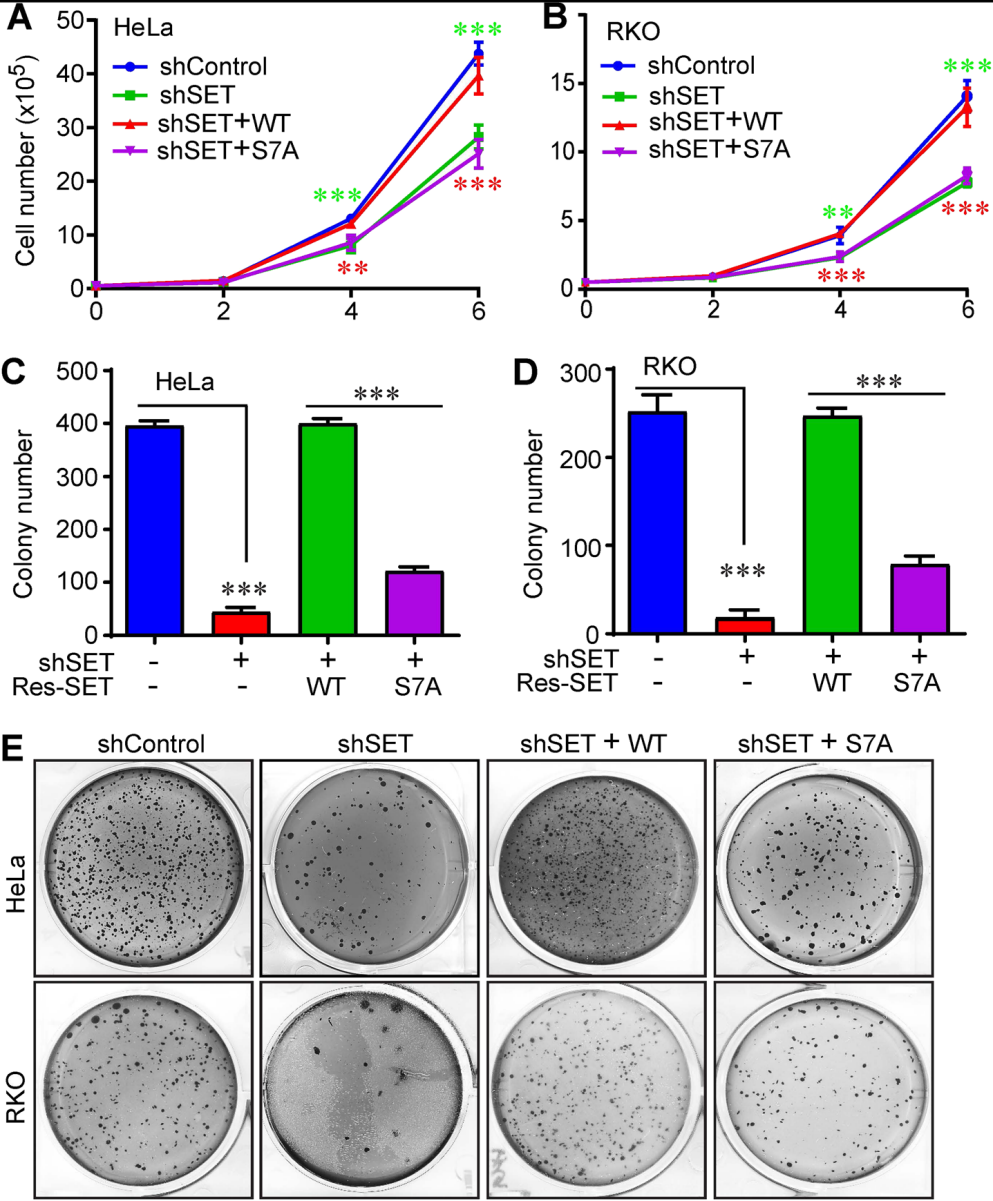
Mitotic phosphorylation of SET isoform 1 is required for cell proliferation and anchorage-independent growth. Mitotic phosphorylation of SET isoform 1 is required for cell proliferation and anchorage-independent growth. **a**, **b** Cell proliferation curves of cell lines established in Figs. 3a (HeLa) and 4c (RKO). Data were expressed as the mean ± SD of three independent experiments. ***p < 0.001; **p < 0.01 (unpaired Student’s *t* test). Green asterisks mark comparisons between shControl and shSET groups. Red asterisks mark comparisons between isoform 1 SET-WT and SET-S7A groups. **c**–**e** Anchorage-independent growth (colony formation assays in soft agar) of cell lines established in Figs. 3a (HeLa) and 4c (RKO). Data were expressed as the mean ± SD of three independent experiments. ***p < 0.001 (unpaired Student’s *t* test)

Consistent with the results in migration and invasion, KD of SET significantly decreased cell proliferation and importantly, expression of wild-type SET isoform 1 completely rescued the cell proliferation defects (Fig. 6a, b). However, cells expressing SET-S7A proliferated at a rate similar to that of SET-KD cells, suggesting that mitotic phosphorylation of SET isoform 1 is required for proper cell proliferation (Fig. 6a, b). Furthermore, SET KD also greatly decreased anchorage-independent growth (colony formation) in soft agar, and again, re-expression of SET-S7A failed to rescue the defects while expression of wild-type SET did (Fig. 6c–e). These data suggest that mitotic phosphorylation is essential for SET isoform 1 to promote cell proliferation and anchorage-independent growth.

### Mitotic phosphorylation of SET isoform 1 is required for its oncogenic activity in vivo

**Fig. 7:**
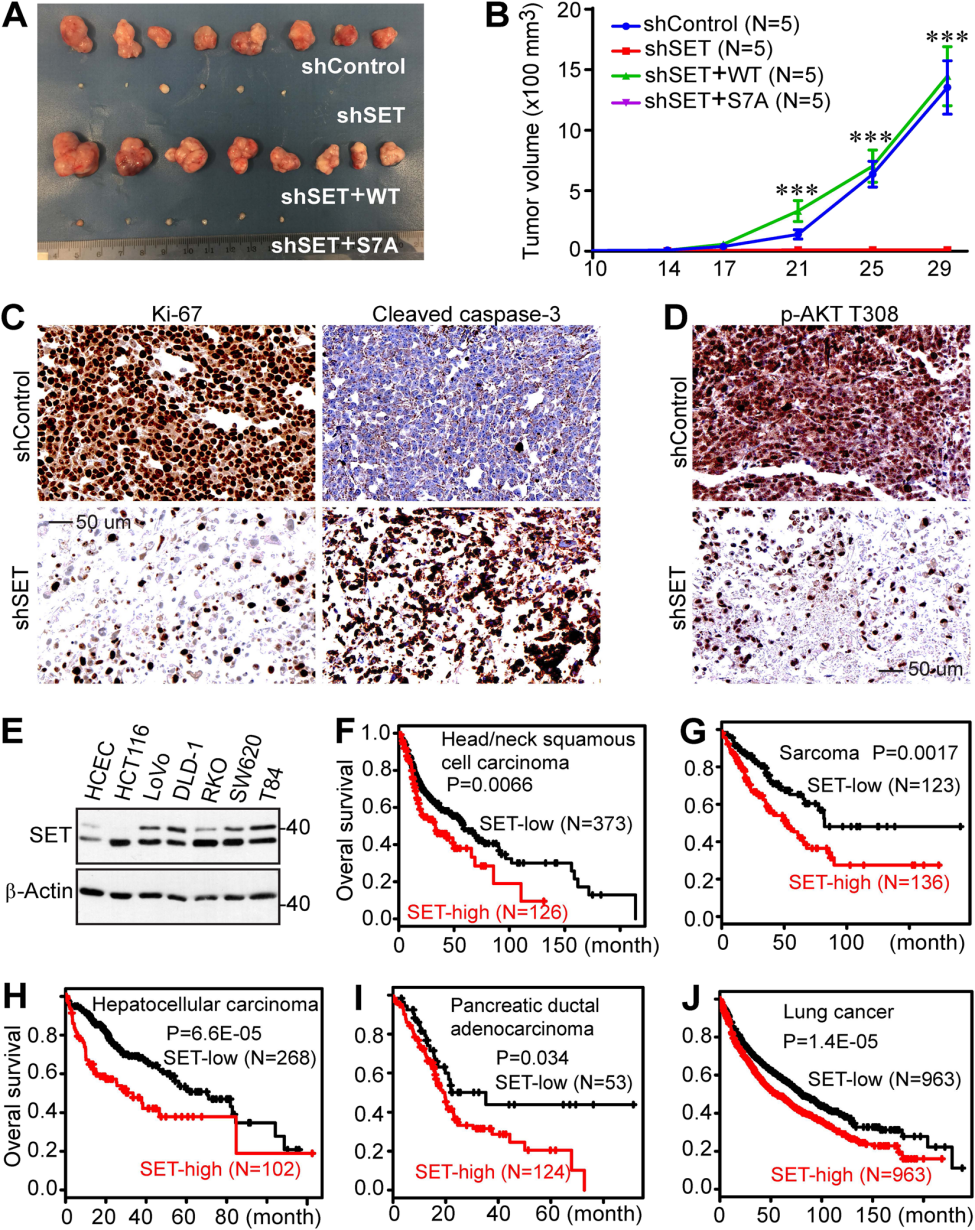
Nonphosphorylated SET isoform 1 is inactive in promoting tumorigenesis in mice. **a** Representative tumors in each group were excised and photographed at the endpoint. **b** Tumor growth curve with various RKO cell lines. Cells were subcutaneously inoculated into athymic nude mice on both flanks (five mice each group). The tumor volume shown at each point was the average from 6 to 10 tumors (two animals in shSET and shSET+SET-S7A groups did not form any palpable tumors, mean ± SD). ***p < 0.001 (unpaired Student’s *t* test). **c** Ki-67 (proliferation) and cleaved caspase-3 (apoptosis) staining in tumors shown in **a**. Tumors from shSET+ isoform 1 SET-WT and shSET+ isoform 1 SET-S7A groups were analyzed. **d** IHC staining with p-AKT T308 antibody in tumors shown in **a**. **e** Western blotting analysis of SET (isoforms 1 and 2) expression in immortalized colon epithelial (HCEC) and colon cancer cell lines. **f**–**j** High expression of SET (all isoforms) correlates with poor clinical outcome. Kaplan–Meier curves of overall survival in various cancers. Data were generated from an online analysis tool (http://kmplot.com) using RNA-seq data^49^

Having demonstrated the role of mitotic phosphorylation of SET in cell culture models, we next evaluated the influence of SET and its mitotic phosphorylation on tumor growth in animals. Various RKO cell lines (Fig. 4c) were subcutaneously inoculated into immunodeficient mice. Interestingly, SET-KD cells formed significantly smaller tumors (if any) when compared with those from control cells (Fig. 7a, b). No significant difference was observed in the sizes of tumors from control and SET-KD cells with exogenous wild-type SET isoform 1. However, cells expressing the isoform 1 SET-S7A mutant formed tumors in size similar to that of SET-KD cells (Fig. 7a, b). Consistent with our observations in Fig. 6, Ki-67 positivity (a proliferation marker) was significantly higher in wild-type SET-expressing tumors than SET-S7A tumors (Fig. 7c). In contrast, massive apoptosis (cleaved caspase-3 staining) was detected and Akt activity (p-AKT T308) was strongly suppressed in SET-S7A tumors (Fig. 7c, d). These results support the hypothesis that mitotic phosphorylation is essential for SET isoform 1-promoted tumor growth in vivo.

Many reports showed that SET (isoforms 1 and 2) is upregulated in cancer cell lines and clinical tumor samples^6^. We found that SET (isoforms 1 and 2) expression is upregulated in colon cancer cells compared with immortalized colon epithelial cells (Fig. 7e). We further analyzed the correlation between SET expression and clinical outcome in published data^49^ and confirmed that high mRNA levels of SET (all isoforms) were significantly correlated with poor survival in various malignancies (Fig. 7f–j).

### Mitotic phosphorylation of SET isoform 1 affects Akt signaling

**Fig. 8:**
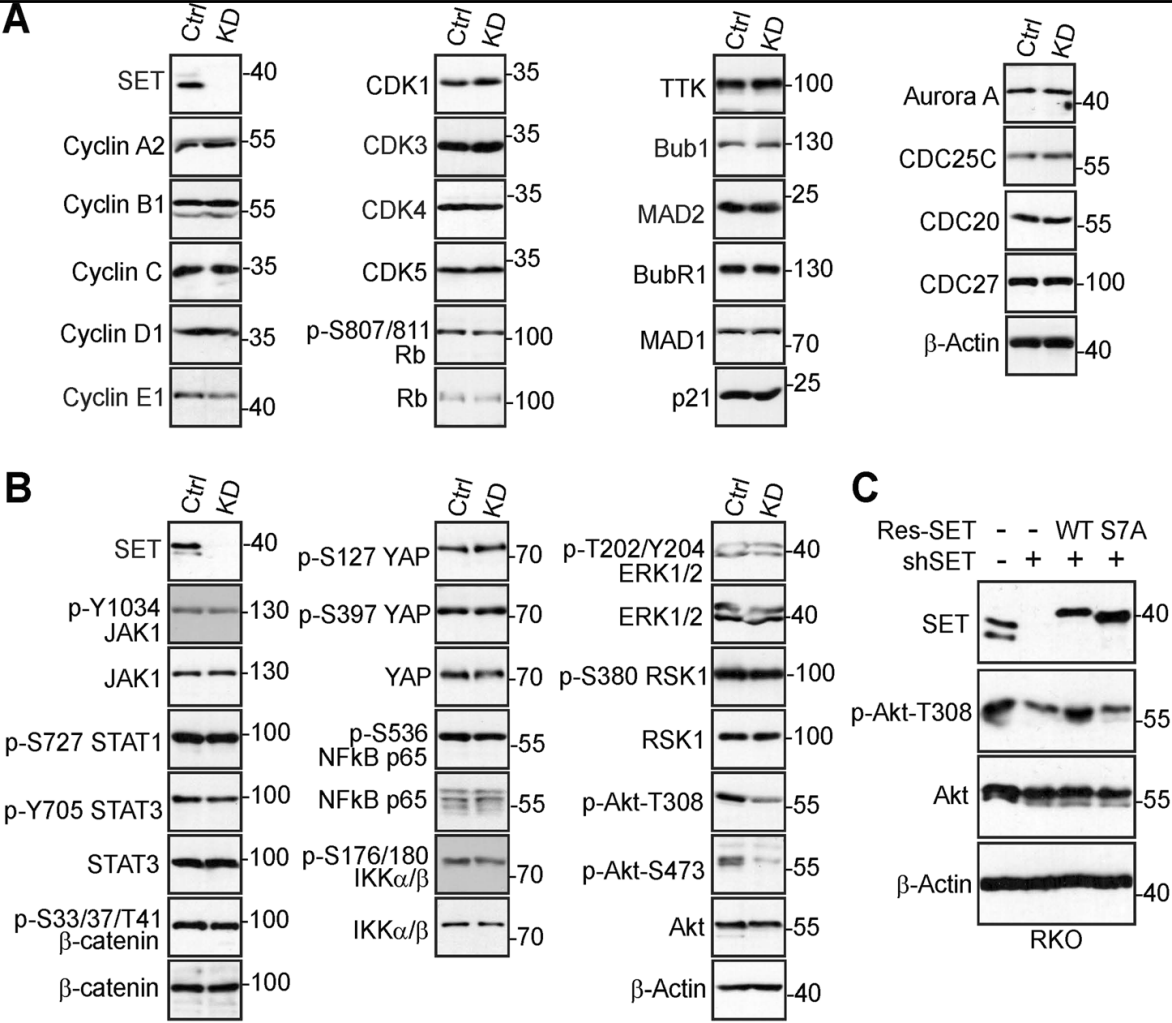
SET isoform 1 phosphorylation affects Akt activity. **a** Expression of cell cycle regulators in shControl (Ctrl) and shSET (KD) HeLa cells. Total cell lysates were probed with the indicated antibodies. **b** Examination of major signaling pathways in control (Ctrl) and SET-knockdown (KD) RKO cells. **c** Western blotting analysis with the indicated antibodies in RKO cells. Data from **a** to **c** are representative of at least two independent experiments

SET regulates cell cycle progression and we hypothesized that one of the underlying mechanisms is through binding to cell cycle regulators^27,28,50^. We surveyed the expression of a panel of cell cycle regulators to determine which one(s) is affected by SET and its mitotic phosphorylation in our system. While most of them were not altered upon SET KD, we found p-S642 WEE1 levels were increased in SET-KD cells (Fig. 8a).

SET has been shown to regulate many signaling pathways, including Wnt/β-catenin, the JAK/STAT, Akt, mitogen-activated protein kinase, etc^6^. To understand the possible involvement of these signaling pathways, we examined their activities in SET KD RKO cells. We found JAK/STAT signaling, nuclear factor kappa-light-chain-enhancer of activated B cells (NF-κB), extracellular-signal-regulated kinase/RSK (ERK/RSK), as well as β-catenin and YAP activities were not affected in SET-KD cells when compared to control cells (Fig. 8b). Consistent with previous studies^51^, Akt activity (revealed by phosphorylation at T308 and S473) was greatly decreased upon SET inhibition in RKO cells (Fig. 8b). Again, re-expression of wild-type SET, but not the SET-S7A mutant, rescued the phenotype (Fig. 8c), suggesting that SET phosphorylation is required for SET-mediated Akt activation (at least via T308 phosphorylation).

## Discussion

Among the two major isoforms of SET, isoform 2 remains at a relatively constant expression level among different tissues and cell lines, and isoform 1 expression varies in a cell- or tissue-specific manner^17,52^. For instance, isoform 1 is absent in some early-stage hematopoietic cell lines and pancreatic cancer cell lines^17,52^. Our current study identifies S7 as a mitotic phosphorylation site, and this modification is essential for its oncogenic activity (Fig. 7). We noted that serine 7 is unique to isoform 1, which raises the question of why some cells/tissues need an additional layer of regulation for SET. Future studies are also needed to determine the biological significance of expression patterns of SET isoforms. Moreover, it will be equally important to elucidate the clinical relevance of CDK1 phosphorylation of SET in future studies. Addressing these questions is anticipated not only to strengthen the comprehension of the biological relevance of CDK1 phosphorylation of SET, but also to be useful in determining potential overlapping and distinguishing functions of the various isoforms of SET during oncogenesis.

SET is also called I2PP2A and interacts with PP2A and another closely related protein I1PP2A/PP32^1–3^. Database analysis (www.phosphosite.org) and phospho-proteomic studies revealed potential mitotic phosphorylation sites at the N-terminus of I1PP2A (T15/S17)^53,54^. The role and regulation of I1PP2A in mitosis have not been defined. We are also interested in investigating how I1PP2A is regulated and whether it also plays a role in mitosis similar to SET/I2PP2A. As an inhibitor of PP2A, SET promotes tumorigenesis mainly through forming an inhibitory protein complex with PP2A^6^. The activity of PP2A is tightly regulated by the C-terminal region of SET that is responsible for the binding to PP2A catalytic subunit PP2A-C^1,2^. Since mitotic phosphorylation occurs on the N-terminus of SET isoform 1, we do not expect that loss of oncogenic activity of SET-S7A is due to alterations in binding with PP2A. Indeed, our coimmunoprecipitation experiments revealed no significant difference between SET and SET-S7A in association with PP2A-C (data not shown). Therefore, it is currently unclear how phosphorylation of SET promotes its oncogenic activity and Akt signaling (Fig. 8). In addition to PP2A, SET has been shown to interact with many other proteins (including CK2, eIF2α, glycogen phosphorylase, TCP1-β, Cyclin B, and p21),^28,50,55^ several of which are involved in mitotic cell cycle progression and tumorigenesis. Does SET phosphorylation affect these binding partners? The future elucidation of these mechanisms will provide novel insights underlying SET-driven tumorigenesis.
